# Surufatinib combined with transarterial embolization versus surufatinib monotherapy in patients with liver metastatic neuroendocrine tumors: Study protocol for a prospective, randomized, controlled trial

**DOI:** 10.1002/cam4.7131

**Published:** 2024-04-17

**Authors:** Ruizhen Li, Xiaofen Li, Xin You, Minggang Su, Yuzhi Liu, Nengwen Ke, Dan Cao

**Affiliations:** ^1^ Department of Abdominal Oncology Cancer Center, West China Hospital, Sichuan University Chengdu China; ^2^ Abdominal Oncology Ward, Division of Medical Oncology Cancer Center, State Key Laboratory of Biotherapy, West China Hospital, Sichuan University Chengdu China

**Keywords:** neuroendocrine tumors with liver metastasis, randomized controlled trial, surufatinib, transarterial embolization

## Abstract

**Background:**

More than half of neuroendocrine tumor (NET) patients will experience liver metastasis, and interventional therapy represented by transarterial embolization (TAE) is the main local treatment method. Surufatinib is recommended as a standard systemic treatment for advanced NETs. The efficacy and safety of surufatinib combined with TAE in the treatment of liver metastasis are undetermined. This study was conducted to compare the clinical outcome of surufatinib combined with TAE versus surufatinib monotherapy in liver metastatic NETs.

**Methods:**

This is a prospective, multicenter, open‐label, and randomized controlled trial. Patients diagnosed with liver metastatic NETs will be enrolled. Participants are randomly assigned in a 1:1 ratio to either the experimental group or the control group. Patients will be treated with surufatinib plus TAE in the experimental group, while patients in the control group will receive surufatinib monotherapy. The primary endpoint is progression‐free survival (PFS) assessed by a blinded independent image review committee (BIIRC). The secondary endpoints are investigator‐assessed PFS, liver‐specific objective response rate (ORR), objective response rate (ORR), disease control rate (DCR), overall survival (OS), and incidence of adverse events.

**Discussion:**

This is the first prospective study to investigate the efficacy of surufatinib combined with TAE. We expect this trial to propose a new and effective treatment strategy for liver metastatic NETs.

## 1 BACKGROUND

Neuroendocrine tumors (NETs) are heterogeneous neoplasms arising in peptidergic neurons and neuroendocrine cells and generally originate in the gastroenteropancreatic (GEP) tract and the lung. In recent decades, the incidence of NETs has significantly increased.^1^, ^2^

The World Health Organization proposed a classification that distinguishes between well‐differentiated NETs and poorly differentiated NECs (NECs) according to different degrees of pathologic differentiation. G1, G2, and G3 grading levels were classified according to the Ki‐67 index (≤2%, 3%–20%, and >20%) and mitotic rates based on the WHO 2010 classifications in gastroenteropancreatic NETs. Although, compared to most neoplasms, NETs have a better prognosis, more than 20% of patients commonly have distant metastases at diagnosis. Previous research has shown that NETs show a relative preference toward the liver irrespective of the primary site, and approximately 82% of all NET patients have liver metastases in the course of disease.^3^, ^4^ Several treatment options exist, including systemic therapy (such as targeted drugs, somatostatin analogues, peptide receptor radionuclide therapy, and chemotherapy) and local therapy (liver resection, transarterial chemoembolization,^5^ and selective internal radiotherapy). Despite such diverse treatment options, there is still uncertainty about the optimal treatment plan. Prospective clinical trials in NETs with liver metastasis have important clinical significance for improving patient prognosis.

Surufatinib, a novel oral tyrosine kinase inhibitor that selectively targets VEGFR 1, 2, and 3, FGFR1, and so on, is being developed for the treatment of NETs. In 2020, two phase III clinical trials (SANET‐p and SANET‐ep) suggested that the median progression‐free survival (PFS) of the surufatinib group was significantly longer than that of the placebo group (pancreatic NETs: 10.9 months vs. 3.7 months, *p* = 0.0011; extrapancreatic NETs: 9.2 months vs. 3.8 months, *p* < 0.0001).^6^, ^7^ 84% of the enrolled patients had liver metastasis, and 66% of the patients had previously received antitumor treatment in the study. The most common treatment‐related adverse events were hypertension, diarrhea, proteinuria, and liver dysfunction. Thus, surufatinib was recommended as standard systemic treatment for locally advanced and metastatic NETs in China.^8^ Based on that NETs hepatic metastases receive most of their blood supply from the hepatic artery, transarterial embolization (TAE) represents a beneficial treatment option for control of symptoms as well as tumor growth,^5^ which is recommended by the guidelines of China and abroad. However, there is no prospective trial to confirm the feasibility of surufatinib plus TAE.^9^, ^10^, ^11^ To investigate this possibility, we planned a prospective study on the efficacy and safety of surufatinib combined with TAE compared to surufatinib monotherapy in patients with liver metastatic NETs, trying to explore a better regimen for NETs with liver metastasis and clinical guidelines.

## 2 METHODS

**FIGURE 1:**
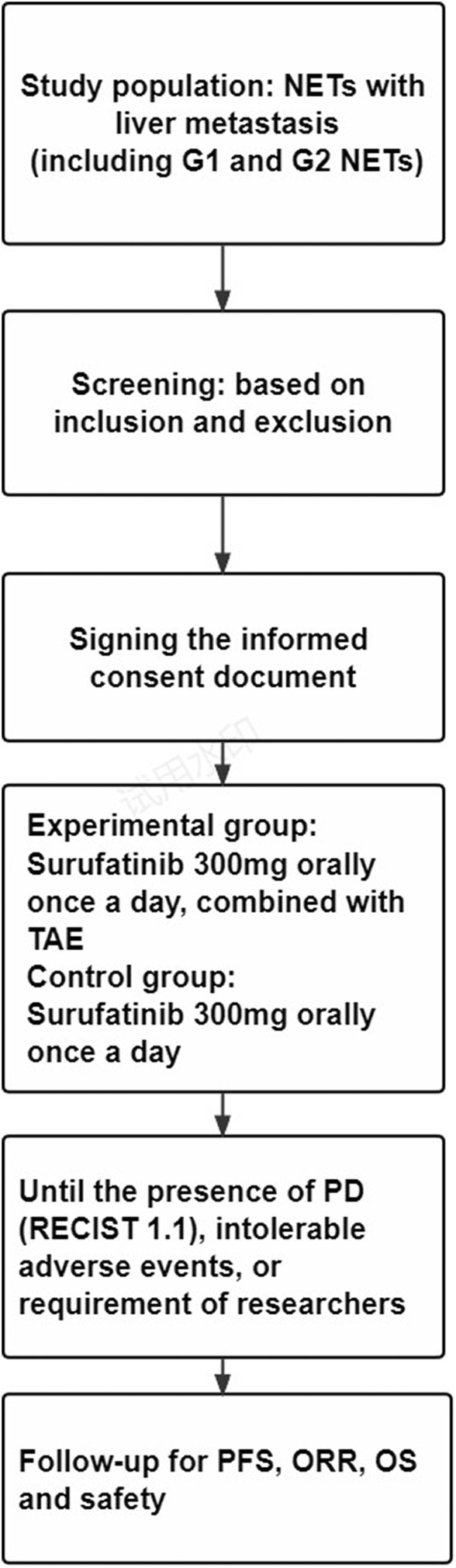
The SPIRIT flow diagram of this trial. NETs, neuroendocrine tumors; TAE, transarterial embolization; RECIST, Response Evaluation Criteria in Solid Tumors; transarterial embolization; PD, progression disease; PFS, progression‐free survival; ORR, objective response rate; DCR, disease control rate; OS, overall survival.

The study is approved by Biomedical Ethics Review Committee of West China Hospital of Sichuan University. The trial is registered at Chinese Clinical Trial Registry (ChiCTR2300071991). The study will be performed at West China Hospital, Sichuan University. Written informed consent will be obtained from all participants. The trial protocol and this manuscript have been developed in line with the Standard Protocol Items: Recommendations for Interventional Trials (SPIRIT) guidelines (Figure 1).^12^

### 2.1 Trial design

This is a prospective, open‐label, and randomized controlled trial. Patients who have been diagnosed with liver metastatic NETs (G1, G2) will be enrolled. Participants are randomly assigned in a 1:1 ratio to either the experimental group or the control group. Patients will be treated with surufatinib plus TAE in the experimental group, while patients in the control group will receive surufatinib monotherapy. This study will prospectively collect progression‐free survival (PFS), liver‐specific objective response rate (ORR), objective response rate (ORR), disease control rate (DCR), overall survival (OS), incidence of adverse reactions, and quality of life (QOL). Unless the patients experience PD or intolerable toxicity, each case should be treated for at least 8 weeks before efficacy evaluation.

## 3 TREATMENT

### 3.1 Experimental group

**FIGURE 2:**
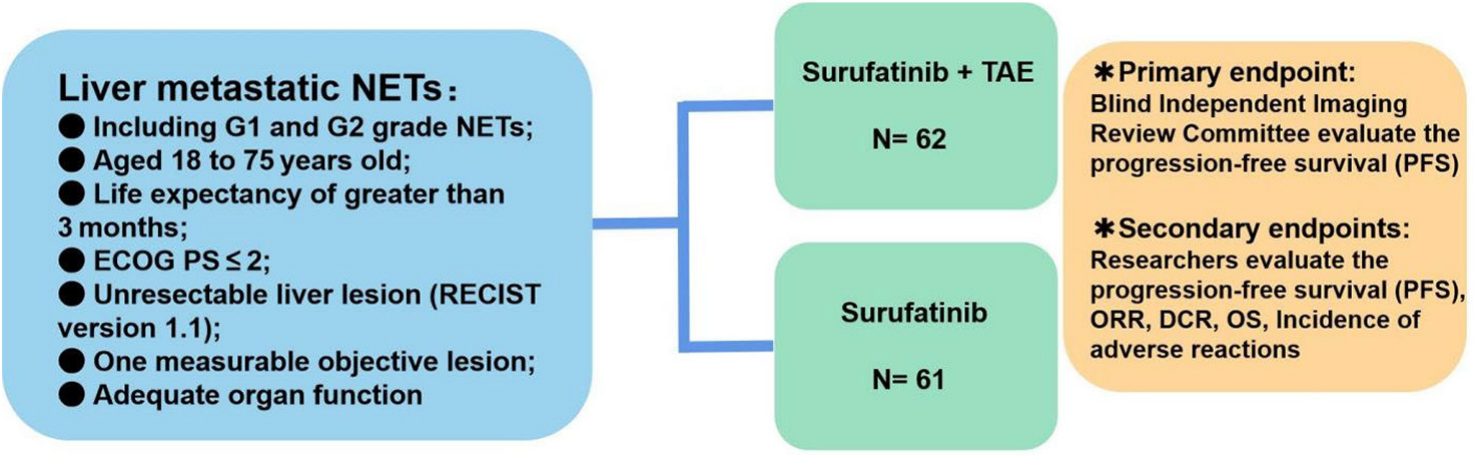
Overview of phases of the trial. ECOG PS, Eastern Cooperative Oncology Group performance status; TAE, transarterial embolization; ORR, objective response rate; DCR, disease control rate; OS, overall survival.

Patients will receive 300 mg of surufatinib orally once a day, combined with TAE. The first TAE treatment should be performed within 14 days after starting surufatinib. During TAE, there is no need to stop surufatinib, and the frequency and interval of TAE should be determined by the interventional physician or multidisciplinary treatment. In the experimental group, patients should receive TAE at least once; for patients with a liver tumor burden exceeding 50%, it is recommended to perform TAE in stages to reduce complications. Each subject will receive treatment until disease progression, intolerable toxicity, or informed consent withdrawal (Figure 2).

### 3.2 Control group

Patients will receive 300 mg of surufatinib orally once a day. Each subject will receive treatment until the PD, intolerable toxicity, or informed consent withdrawal (Figure 2).

## 4 ENDPOINTS

### 4.1 The primary endpoint

The PFS was assessed by a blinded independent image review committee (BIIRC).

### 4.2 The secondary endpoints

Investigator‐assessed PFS, liver‐specific objective response rate (ORR), ORR, disease control rate (DCR), overall survival (OS), and incidence of adverse events.

## 5 INCLUSION AND EXCLUSION CRITERIA

### 5.1 Inclusion criteria

1. Histologically or cytologically confirmed G1/G2 NETs with liver metastasis; the location of primary lesions and whether surgical resection is performed are not limited;
2. Aged 18–75 years old;
3. Life expectancy of longer than 3 months;
4. Eastern Cooperative Oncology Group performance status score (ECOG) ≤2;
5. According to imaging and surgical evaluation, the liver lesion is unresectable or the patient is unable to tolerate surgery, and the primary lesion has no serious complications (perforation, obstruction or hemorrhea that cannot be managed by medical therapy);
6. The liver must have at least one measurable objective tumor lesion according to the RECIST version 1.1 standard, with a maximum diameter of ≥1 cm for spiral CT examination and ≥2 cm for conventional CT or MRI, and it should be conducted within 28 days before enrollment.
7. Adequate organ functions as follows:

Absolute neutrophil count ≥1500/mm^3^, leukocyte≥3000/mm^3^, platelet count ≥75,000/mm^3^, hemoglobin ≥9.0 g/dL; total bilirubin≤1.5 × upper limit of normal (UNL); serum creatinine ≤1.5 × UNL; alanine aminotransferase, aspartate aminotransferase ≤5 × UNL;

8 Participate the study voluntarily and sign the informed consent document.

### 5.2 Exclusion criteria

1. Histologically or cytologically confirmed neuroendocrine carcinoma, small cell carcinoma, and goblet cell carcinoid;
2. Functional neuroendocrine tumors that require long‐acting somatostatin analogues (SSAs) to control symptoms, such as islet cell tumors, glucagonoma, gastrinoma, vasoactive intestinal polypeptide‐secreting tumors, adrenocorticotropic hormone‐secreting tumors, and carcinoid syndrome;
3. Urine protein: ≥2+ or >1.0 g/24 h;
4. Patients with hypertension (> 140 mmHg systolic or >90 mmHg diastolic) that cannot be adequately controlled with antihypertensive agents;
5. Patients with serious gastrointestinal diseases that may affect drug absorption, including but not limited to the presence of a pepticulcer, ulcerative colitis, active gastrointestinal bleeding, gastrointestinal obstruction, and severe diarrhea;
6. Patients with a history of severe hemorrhagic disease (single blood loss >30 mL) within 3 months, hemoptysis (single blood loss >5 mL) within 1 month, or thromboembolic events (including pulmonary embolism and cerebral infarction) within 12 months;
7. Patients received surgical treatment (excluding biopsy) within 4 weeks or the surgical incision healed in poor condition;
8. Patients with severe cardiovascular diseases, including but not limited to acute myocardial infarction, unstable angina, heart failure, ventricular arrhythmias requiring medication, and left ventricular ejection fraction <50%, a corrected QT interval of ≥480 ms on ECG;
9. Patients with other malignant tumors with a disease‐free survival <5 years (except for cured basal cell carcinoma of the skin, cured cervical in situ carcinoma, and cured gastrointestinal tumors through endoscopic mucosal resection);
10. Patients with immune deficiency diseases or HIV infection;
11. Patients with severe uncontrolled acute infection (infection causing fever above 38 centigrade);
12. Patients with active hepatitis B or active hepatitis C, and hepatitis B virus (HBV) DNA ≥ 1 × 10^4^ IU/mL, and hepatitis C virus (HCV) RNA≥1 × 10^3^ IU/mL;
13. Patients with other serious medical and surgical diseases and the investigator believes that it is not suitable to participate in this clinical trial;
14. Patients who participated in clinical trials of other novel drugs within 4 weeks;
15. Pregnant or lactating women or patients with fertility (male or female with less than 1 year of amenorrhea) are unwilling to take contraceptive measures;
16. Patients who have severe study drug allergy and hypersensitivity;
17. Unsuitable for enrollment in the opinion of the investigator.

### 5.3 Assessment and follow‐up

The comprehensive information of eligible patients needs to be collected at least 1 week before the start of treatment, which includes: medical history, physical examination, ECOG PS score, the test of clinical chemistry, hematology and coagulation, electrolytes, liver, kidney, and thyroid function, CA 199, CA 125, CEA, NSE, CT or MRI scan, and ECG. Disease evaluation is performed every 12 weeks by CT or MRI based on RECIST version 1.1. Whether CT or MRI is used is up to the investigator, but the evaluation method, machine, and technical parameters should be kept as consistent as possible throughout the entire treatment. The imaging examination window is ±7 days. The disease condition and survival status will be recorded via face‐to‐face visits or by phone every 4 weeks. After the first evaluation, patients with SD, PR, or CR continued treatment, while PD patients were excluded from the study.

### 5.4 Dosage adjustments

Toxic reactions should be classified based on NCI‐CTC AE version 5.0. Dosage adjustments of surufatinib should be guided by toxicity grading criteria. General principles include:

1. For adverse reactions, appropriate treatment should be administered to alleviate patient symptoms and signs. This may involve administering anti‐hypertensive drugs for hypertension and hepatoprotective drugs for liver function abnormalities.
2. Pre‐existing adverse events at baseline, if deemed necessary by the researcher, will be adjusted according to changes in toxicity grading.
3. In cases of multiple simultaneous adverse reactions, the most conservative dose adjustment plan should be implemented.
4. Following adverse reactions, surufatinib dosage should not be re‐increased, and dosage should not be reduced more than three times during each patient's treatment course.
5. The maximum duration of medication suspension due to toxic reactions is 4 weeks. If the adverse reaction decreases to ≤1 level within this period, dosage adjustment to resume medication is recommended. Specific dosage adjustment principles are outlined in the drug instructions. The principles of dosage adjustment are presented in Table 1.
6. If researchers determine that a toxic reaction is unlikely to escalate to a serious or life‐threatening event, such as a rash, patients may continue treatment at the original dose without reduction or interruption. In the case of non‐hemolytic anemia, if better relief can be attained through interventions like blood transfusion, erythropoietin, and iron therapy, dosage reduction or treatment interruption may be unnecessary.

**TABLE 1:** Dose adjustment of surufatinib.

Dose adjustment gradients	
Original dose of surufatinib	300 mg qd po, q4w
The first reduction	250 mg qd po, q4w
The second reduction	200 mg qd po, q4w
The third reduction	200 mg/250 mg d1‐d21 po, q4w

**General principles of dose adjustment**	
Grade 1–2	No adjustment required
Bleeding from any site classified as grade ≥2; Creatinine levels exceeding twice the upper limit of normal or baseline value but not exceeding three times the upper limit of normal or baseline value.	Suspension of surufatinib; Recovery to ≤ grade 1 within 4 weeks requires a reduction in dose level by one dose level
Grade 3 or higher bleeding; gastrointestinal perforation; nephrotic syndrome or hypertensive crisis; arterial thrombosis.	Permanent cessation of surufatinib
Other Grade 3 or 4 AE	Suspension of surufatinib; Recovery to ≤ grade 1 within 4 weeks requires a reduction in dose level by one dose level

### 5.5 Sample size and statistical analyses

Referring to the results of two phase III clinical studies of surufatinib (SANET‐p and SANET‐ep), the sample size calculation was performed using the log rank method on PASS, and the hazard ratio (HR) was set as 0.6 with a β error of 0.2 and a two‐sided α level of 10%. Patients were randomly assigned to the experimental group and control group in a 1:1 ratio, with an estimated dropout rate of 5% for each group. The study will last for 3 years, and the enrollment of subjects will occur in the first 2 years. Therefore, it is estimated that at least 62 patients are needed in the experimental group and 61 patients in the control group. A total of 123 patients and 96 events are needed in both groups.

The primary objectives are to determine BIRC‐assessed PFS. The secondary objectives are to determine investigator‐assessed PFS, OS, ORR, and safety. PFS is defined as the time from the beginning of treatment until the first observed disease progression or cancer‐related death. OS will be evaluated from the start of treatment to the death of patients. In this trial, ORR is defined as CR + PR according to RECIST 1.1. The 95% confidence intervals (CI) of the ORR will be calculated. The Kaplan–Meier method and a two‐tailed log rank test are used to evaluate PFS and OS.

## 6 DISCUSSION

This trial aims to explore the efficacy and safety of surufatinib combined with TAE compared to surufatinib monotherapy. SANET‐p and SANET‐ep trials have demonstrated that surufatinib significantly improves median PFS in patients with pancreatic and extrapancreatic NETs compared to placebo.^6^, ^7^ Therefore, surufatinib has been recommended by the guidelines as the standard treatment for advanced NETs.^8^

Due to the abundant blood supply of liver lesions, interventional therapy represented by transarterial embolization (TAE) is the main local treatment because it cannot be surgically eradicated.^5^ The phase 3 trial, LAUNCH study,^13^ indicated that in hepatocellular carcinoma, tyrosine kinase inhibitors (TKIs) combined with TAE showed significant survival improvement compared to TKIs monotherapy. TKIs combined with TAE in primary hepatic neuroendocrine tumors have been reported in some cases,^14^ and patients have maintained a good efficacy. Previous study demonstrated that tumor debulking with TAE may improve the efficacy of systemic TKIs treatment and allow longer PFS.

This study is the first prospective trial to compare the efficacy of surufatinib plus TAE versus surufatinib monotherapy to find new treatment strategies for patients with liver metastatic NETs, which is expected to improve prognosis and provide an important reference for clinical therapy and guideline development.

## AUTHOR CONTRIBUTIONS

**Ruizhen Li:** Data curation (equal); formal analysis (equal). **Xiaofen Li:** Resources (equal). **Xin You:** Investigation (supporting). **Minggang Su:** Investigation (supporting). **Yuzhi Liu:** Investigation (supporting). **Nengwen Ke:** Investigation (supporting). **Dan Cao:** Conceptualization (lead); funding acquisition (lead).

## FUNDING INFORMATION

This work was supported by the project for disciplines of excellence‐Clinical Research Incubation Project, West China Hospital, Sichuan University.

## CONFLICT OF INTEREST STATEMENT

The authors declare no conflicts of interest.

## Data Availability

I confirm that I have included a citation for available data in my references section, unless my article type is exempt.
